# Preparation and Performance Study of Uniform Silver–Graphene Composite Coatings via Zeta Potential Regulation and Electrodeposition Process Optimization

**DOI:** 10.3390/nano15191523

**Published:** 2025-10-05

**Authors:** Luyi Sun, Hongrui Zhang, Xiao Li, Dancong Zhang, Yuxin Chen, Taiyu Su, Ming Zhou

**Affiliations:** 1 School of Mechanical and Automotive Engineering, Guangxi University of Science and Technology, Liuzhou 545006, China; 2 Guangxi Jianxing Guangyin New Material Technology Co., Ltd., Nanning 530024, China; 3 Yingcheng Vocational Education Center, Yingcheng 432400, China; 4 Guangxi Tsinglube New Material Technology Co., Ltd., Liuzhou 545006, China

**Keywords:** zeta potential, dispersion control, double-pulse electrodeposition, composite coatings, performance studies

## Abstract

High-performance electrical contact materials are crucial for electric power systems, new energy vehicles, and rail transportation, as their properties directly impact the reliability and safety of electronic devices. Enhancing these materials not only improves energy efficiency but also offers notable environmental and economic advantages. However, traditional composite contact materials often suffer from poor dispersion of the reinforcing phase, which restricts further performance improvement. Graphene (G), with its unique two-dimensional structure and exceptional electrical, mechanical, and tribological properties, is considered an ideal reinforcement for metal matrix composites. Yet, its tendency to agglomerate poses a significant challenge to achieving uniform dispersion. To overcome this, the study introduces a dual approach: modulation of the zeta potential (ζ) in the silver-plated liquid to enhance G’s dispersion stability, and concurrent optimization of the composite electrodeposition process. Experimental results demonstrate that this synergistic strategy enables the uniform distribution of G within the silver matrix. The resulting silver–graphene (Ag-G) composite coatings exhibit outstanding overall performance at both micro and macro levels. This work offers a novel and effective pathway for the design of advanced electrical contact materials with promising application potential.

## 1. Introduction

In key areas of manufacturing, such as aerospace, electric power equipment and new energy vehicles, electrical contact materials, as the core components ensuring the stable operation of electronic systems, are critical to the overall reliability of the equipment by optimizing their performance [1]. These materials face many challenges under current-carrying friction conditions: high electrical conductivity, low contact resistance, resistance to arc welding and excellent wear resistance need to be balanced. However, with the development of equipment miniaturization and intelligence, it is difficult for traditional materials to meet the requirements of the synergistic optimization of these properties. In critical applications such as rail transportation and smart grids, the performance of electrical contact materials directly affects the reliability and safety of the system [2,3]. Especially under current-carrying conditions, the friction chemical reaction film formed on the material surface can significantly change the electro-mechanical properties of the contact interface [4]. Therefore, there is an urgent need for in-depth research on the evolution mechanism of material interfaces and the development of novel composites and surface modification techniques to achieve synergistic enhancement of electrical conductivity, mechanical properties, and tribological properties.

Silver-based electrical contact materials occupy an important position in the fields of power electronics and automation control due to their excellent electrical conductivity (low resistivity and contact resistance) [5,6]. However, pure silver materials have obvious defects: they have low hardness, have poor wear resistance, are easily subject to wearing under mechanical friction conditions; in sulfur-containing environments, silver surfaces are prone to the formation of silver sulfide films, resulting in a sharp increase in the contact resistance, which seriously affects the reliability of equipment [7,8]. To address these issues, researchers have explored enhancing material properties by introducing secondary phases such as graphite, molybdenum disulfide, and nickel into metal matrices. Although these additions can improve tribological performance, they often compromise electrical conductivity [9,10,11]. To break through this bottleneck, Wang et al. [12] innovatively synergistically introduced carbon nanotubes (CNTs) and graphite into the silver matrix and successfully prepared CNTs-Ag-graphite ternary composites using a powder metallurgical process. This novel material exhibits significant advantages in tribological properties, with the carbon nanotubes inducing the graphite to form a more stable lubrication transfer film, which significantly reduces the friction coefficient and wear rate; while the excellent electrical conductivity of the carbon nanotubes effectively compensates for the loss of electrical conductivity brought about by the graphite. Although the performance of the material has been improved, there is still a gap between its conductivity and that of the pure silver material, which points to the direction of improvement for subsequent research.

Graphene (G), as an emerging two-dimensional material, has excellent electrical, mechanical, and tribological properties [13,14,15]. Experiments have confirmed that the introduction of trace amounts of G can significantly enhance the comprehensive properties of the base metal, including mechanical properties such as hardness and modulus of elasticity [16], yield strength [17], and flexural strength [18], as well as physicochemical properties such as abrasion resistance [19], corrosion resistance [20], thermal conductivity [21], and electrical conductivity [22]. Yang et al. [23] investigated the effect of G content on the mechanical and corrosion properties of aluminum-based composites. The results showed that a G content of 0.3 wt% resulted in the highest tensile strength and the lowest coefficient of friction. Mao et al. [24] investigated sliding silver-based electrical contact materials, focusing on their tribological properties and electrical conductivity under G lubrication. It was found that G could form a long-life protective film on the friction surface, resulting in significant reductions in friction and wear, and contributing to an extended service life of the electrical contact materials. Meanwhile, the composite film formed by G and silver has a lower contact resistance than that of the pure silver film. Lv et al. [25] investigated the properties of silver (Ag), silver–graphite (Ag-C) and silver–graphene (Ag-G) coatings, revealing how G enhances silver matrix composites. In the Ag-G coating, G improved the tribological properties and corrosion resistance of the materials by refining the metal grains and restricting the diffusion of electrons and ions at the grain boundaries, while maintaining the electrical conductivity similar to that of pure Ag coating. Recent findings suggest that the uniform distribution of G in the matrix is critical to the performance of the composites [26,27,28,29]. Therefore, how to effectively improve the homogeneous distribution of G in the matrix has become a key issue to be solved.

Electrodeposition serves as the core preparation technique in this study, demonstrating significant advantages in the synthesis of functional materials [30]. Based on electrochemical principles, this method enables the deposition of materials with excellent contour-following capability, uniform density, and pinhole-free properties on complex substrates. It is suitable for the efficient preparation of metals, polymers, and nanocomposites [31]. For instance, by adjusting electrochemical parameters, it enables the controlled synthesis of gradient polymer films [32] or metal oxide semiconductors [33]. Electrodeposition can also be employed to construct transparent nanocomposite films based on metal clusters [34]. In the preparation of metal–graphene composites, compared to traditional powder metallurgy and stirred casting processes [35,36] that require high-temperature treatment and may cause irreversible damage to graphene structures, studies indicate that 500 °C can already induce significant defects in single-layer graphene [37]. Composite electrodeposition technology, with its low operating temperature, effectively avoids high-temperature damage. Moreover, it enables the synergistic and uniform deposition of graphene and metal ions through a liquid-phase environment, making it an ideal choice for fabricating high-performance composite materials.

Aiming to mitigate the issue of G being prone to agglomeration, leading to poor dispersion in the metal matrix, our team previously introduced a material design strategy that leverages π–π interactions between G and conjugated compounds to facilitate its uniform dispersion within the metal matrix, thereby improving the overall composite performance. Guided by this approach, Ag-G composite coatings were fabricated on copper substrates through an electrodeposition process, employing environmentally benign nicotinic acid as a cyanide-free complexing agent [38]. To overcome the bottlenecks of agglomeration of reinforcing phases and limited performance enhancement in existing silver–graphene composite electrodeposition techniques (such as the direct current plating method employed in Lv et al.’s 2021 study [25]), this study innovatively combines graphene surface modification with a dual-pulse electrodeposition process. By synergistically regulating the zeta potential of the plating solution and pulse electrical parameters, it achieves highly uniform graphene distribution while effectively refining the silver matrix grains. Performance characterization reveals that this synergistic optimization strategy significantly enhances the coating’s comprehensive properties, manifested in markedly increased microhardness, markedly reduced surface roughness, and simultaneously improved wear resistance and corrosion resistance. These performance enhancements hold clear engineering value for electrical contact applications: increased hardness enhances the coating’s resistance to plastic deformation, while the smooth surface ensures stable contact conditions. The synergistic effect of these properties enables the coating to exhibit excellent contact resistance stability during long-term electrical contact testing. This study confirms that the strategy effectively enhances the arc erosion resistance of electrical contacts and extends their service life, providing a practical solution for developing high-reliability electrical contact materials.

## 2. Materials and Methods

### 2.1. Material Characterization and Pretreatment

#### 2.1.1. Material Properties

The base material of most electrical contact materials is pure copper, so in this study, T_2_ copper violet plate with the size of 50 mm × 50 mm × 0.5 mm was used as the cathode, which was purchased from Taizhou Thousand Interest Metal Materials Co., Ltd. (Taizhou, China). A silver plate with a size of 25 mm × 40 mm × 2 mm and a purity of ≥99.9% was used as the anode, purchased from Shenzhen Guoyin Tongbao Co., Ltd. (Shenzhen, China). Graphene (G) and graphene oxide (GO) were used as the reinforcing phase, purchased from Shenzhen Suiheng Graphene Technology Co., Ltd. (Shenzhen, China). The specifications of G were as follows: few layers, sheet diameter 7–12 μm, carbon content ≥ 98%, black powder. Specification parameters of GO: few layers, sheet diameter of 5–10 μm, brown powder. Silver nitrate, ammonium acetate, anhydrous potassium carbonate, potassium hydroxide and ammonia solution were purchased from Xilong Science Co., Ltd. (Shantou, China). Nicotinic acid, sodium chloride, 5,5-dimethylhydantoin, sodium dodecyl sulfate (SDS), sodium dodecylbenzene sulfonate (SDBS), and polyvinylpyrrolidone (PVP) were purchased from Shanghai Macklin Biochemical Co., Ltd. (Shanghai, China).

#### 2.1.2. Preprocessing

This study uses copper substrate as the experimental substrate, its pretreatment process mainly includes three key steps: firstly, surface cleaning treatment, degreasing treatment; secondly, through the tape molding to leave a 50 mm × 10 mm silver-plated work area, and in turn with P2000, P3000, P4000, P5000 sandpaper gradient sanding of the area to the mirror bright, and supplemented with ultrasonic cleaning with pure water; Finally, pre-silver plating treatment, rinsed and dried by deionized water and then prepared for use.

### 2.2. Preparation of Coating

**Figure 1:**
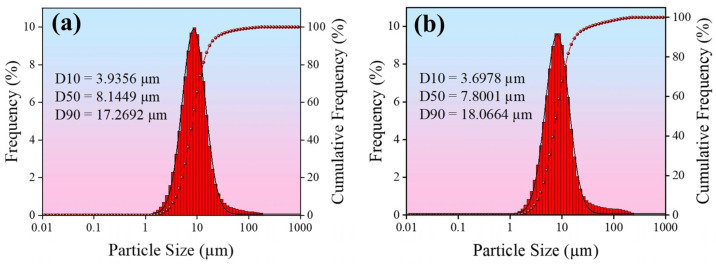
Particle size histogram in the plating solution: (**a**) G; (**b**) GO.

In this study, electrodeposition was carried out using a double-pulse power supply (Shanghai, China) with a high-purity silver plate (purity ≥ 99.9%) as the anode and a pre-treated copper violet substrate as the cathode. The electrodeposition was carried out in a standard glass beaker with a cathode-anode spacing of 5–7 cm, and the temperature of the plating solution was maintained at 30 °C. The stirring speed was controlled at 260 rpm using a collector-type thermostatically heated magnetic stirrer (Gongyi, China). The plating solution composition and plating parameters of the four coatings prepared in this study are shown in Table 1, of which, #1 represents a pure silver coating obtained via direct current electroplating using a pure silver plating solution, serving as the control group; #2 and #3 coatings were prepared using direct current electroplating with silver graphene (Ag-G) and silver oxide graphene (Ag-GO) plating solutions at a concentration of 0.75 g/L, respectively; #4 The coating was prepared using an optimized dual-pulse electroplating process based on a 0.75 g/L Ag-GO plating solution. Figure 1 shows the particle size distribution of G and GO in the plating solutions. The median diameters of both are relatively close, consistent with the parameters of the aforementioned G and GO powders. In comparison, GO exhibits slightly smaller particle sizes, resulting in better dispersion.

### 2.3. Characterization and Testing Methods

#### 2.3.1. Characterization Methods

In this study, several advanced characterization techniques were used to fully analyze the coatings. Surface micromorphology and roughness were obtained using an OXFORE-Cypher ES atomic force microscope (AFM, Oxford, UK) (BRUKER TAP525A probe) in the tap mode; the surface and cross-section morphology of the coatings were observed by a ZEISS-SIGMA 300 scanning electron microscope (SEM, Portland, OR, USA) and analyzed for elemental distribution in conjunction with energy dispersive spectrometer (EDS, Portland, OR, USA); and using a BRUKER-D8 Advance X-ray diffractometer (XRD, Karlsruhe, Germany) (Cu Kα radiation source) in the range of 2θ = 30–85° to collect the diffraction data and calculate the grain size based on the Debye-Scherrer formula; the structural characteristics of G were characterized by HORIBA-XploRA plus Raman spectrometer (Kyoto, Japan); finally, the surface was quantitatively analyzed by a BRUKER-NPFLEX 1000 white-light interferometric three-dimensional topography scanner (Karlsruhe, Germany) roughness and wear volume.

#### 2.3.2. Test Methods

In this study, the performance of the composite coatings was systematically evaluated using a multiscale testing method. Firstly, ζ of the silver plating solution was measured by HORIBA-SZ-100V2 Zeta Potentiostat (Kyoto, Japan), and 10 replicate measurements were performed for each sample, and the average of 8 data was taken after eliminating the extreme values, in order to evaluate the dispersion stability of G in the plating solution. For the mechanical property test, HV-1000 Vickers hardness tester (Laizhou, China) (load 0.2 kgf, hold time 10 s) was used to measure the microhardness of the coatings, and 10 test points were selected for each sample and the average of valid 8 points was taken. Tribological properties were evaluated by BRUKER-Tribolab friction and wear tester (UMT, Karlsruhe, Germany), under the conditions of load 5 N, frequency 3 Hz, stroke 3 mm, GCr15 ball with diameter 6.35 mm was used as the counter-abrasion vice for 20 min test. During the current-carrying friction tests, a constant current of 5 A was applied. Real-time contact resistance measurements were recorded using a multimeter, with each sample tested three times to obtain an averaged result. Prior to testing, all samples were polished uniformly to ensure consistent surface roughness (P7000 sandpaper). Corrosion resistance was tested using CHI660E electrochemical workstation (Ningbo, China) for kinetic potential polarization test, and the three-electrode system was used to obtain the Tafel curve in 3.5% NaCl solution at a scanning rate of 5 mV/s, and the electrochemical parameters were calculated by extrapolation method. The electrical contact life test was performed using a JF04D tester (Kunming, China) (DC 24 V/5 A, contact pressure 40 cN, spacing 1 mm, frequency 1 Hz, duty cycle 50%), and the contact resistance was recorded every 100 actions. This comprehensive test program comprehensively tests the performance characteristics of the composite coating from multiple dimensions such as dispersion stability, mechanical properties, friction and wear characteristics, corrosion resistance and electrical contact reliability.

## 3. Results and Discussion

### 3.1. Modulation of Zeta Potential in Silver-Plated Liquid Systems

This study firstly focuses on the dispersion stability of G in the process of composite electrodeposition, and establishes a complete G dispersion optimization scheme by systematically regulating the ζ of the plating solution based on the nicotinic acid cyanide-free silver plating system. Two strategies of non-covalent modification and covalent modification were used in the study, and the mechanisms of non-covalent modification and covalent modification on the stability of the plating solution were deeply investigated.

**Figure 2:**
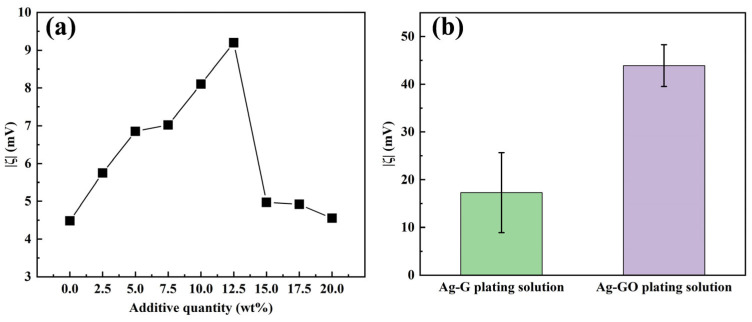
(**a**) The relationship between the |ζ| value and the amount of SDS added; (**b**) |ζ| values in Ag-G and Ag-GO composite electroplating solutions.

In non-covalent modification, G improves dispersibility and interfacial compatibility by physically adsorbing surfactants and water-soluble polymers without altering its chemical structure. Their amphiphilic nature allows hydrophobic groups to adsorb onto the G surface via van der Waals forces or π–π interactions, while hydrophilic groups face the solvent. Concurrently, the long-chain structures of water-soluble polymers create steric hindrance, thereby forming a stable dispersion system. For the strongly alkaline environment of electroplating solutions, alkali-resistant surfactants sodium dodecyl sulfate (SDS) and sodium dodecylbenzene sulfonate (SDBS) were selected, along with the water-soluble polymer polyvinylpyrrolidone (PVP). The effects of different additions of SDS (as a percentage of the weight of G) on the ζ of Ag-G composite plating solution (1 g/L) were systematically investigated. The results showed that the optimal addition amount of SDS was 10–12.5 wt% (as shown in Figure 2a). Based on the quantitative analysis results of the surfactants mentioned above, an orthogonal experimental design (L_9_(3^4^)) was further employed to systematically investigate the interactions among the three additives in their composite formulation. The data in Table 2 show that when the addition ratios of SDS, SDBS, and PVP are 4 wt%, 5 wt%, and 3 wt%, respectively, the absolute value of the zeta potential (|ζ|) of the Ag-G composite plating solution reaches its maximum value, indicating that the dispersion stability of the silver plating solution system is optimal at this ratio. Then, range analysis was performed on the experimental results to further reveal the order of preference for each additive’s effect on the zeta potential. The range analysis in Table 2 shows that the order of preference for ζ is PVP > SDS > SDBS, and based on the range analysis, the preferred process conditions are: 4 wt%, 3 wt%, and 3 wt% for the additions of SDS, SDBS, and PVP, respectively. Finally, we also compared the preferred solutions derived from direct analysis and range analysis for experimental testing and comparison, and finally determined that 4 wt% SDS, 5 wt% SDBS, and 3 wt% PVP were the optimal compounding ratios, this resulted in a value of 17.29 mV for |ζ| in the Ag-G composite electroplating solution.

In terms of covalent modification, GO is the product of covalent modification of G by chemical oxidation, which is a typical covalent modification process [39]. In this study, GO was used as the reinforcing phase, and the hydrophilic functional groups, such as -COOH and -OH, enriched on its surface, significantly increased the |ζ| of the Ag-GO plating solution (1 g/L) to 43.90 mV by the dual mechanism of electrostatic displacement repulsion and hydrogen bonding, which is a 2.5-fold increase compared with the non-covalently modified system (Figure 2b). It has been shown that GO can be electrochemically reduced to G in situ during the electrodeposition process, which maintains the intrinsic properties of G and enhances the interfacial bonding through surface functional groups, effectively solving the problem of G agglomeration [40]. Therefore, covalently modified G was preferred as the reinforcing phase added in the plating solution in this study.

### 3.2. Optimization of Coating Preparation Process

Aiming at the difficulty of dispersion of G in the substrate due to its easy agglomeration, the innovative double-pulse composite electrodeposition technique was adopted to realize the precise regulation of G dispersion in the coatings and the coating properties through its unique bidirectional current characteristics (forward deposition/negative dissolution). The study adopted an orthogonal experimental design (L_16_(4^5^)) combined with a single-factor test method to systematically investigate the effects of five key process parameters, namely GO concentration (C_GO_), forward average current density (J_f_), negative average current density (J_r_), forward duty cycle (D_f_), and negative duty cycle (D_r_), on coating performance. The results of the orthogonal tests and the range analysis are shown in Table 3 and analyzed as follows.

Firstly, based on the test results of microhardness (HV) and average friction coefficient (COF) in the orthogonal test, the optimal parameter combinations for the electrodeposition process of Ag-G composite coatings can be determined by direct comparative analysis as follows: the concentration of GO is 1.0 g/L, the average forward current density is 0.50 A/dm^2^, the average negative current density is 0.08 A/dm^2^, the forward duty cycle is 70.0%, and the negative duty cycle is 30.0%, as shown in Table 3, sample 8. The Ag-G composite coating prepared under this parameter has a microhardness of 141.813 HV_0.2_, which corresponds to an average coefficient of friction of 0.510, showing high mechanical and tribological properties.

The results of range analysis in Table 3 show that: when taking microhardness as the evaluation index, the priority order of the influence of each parameter on the microhardness of the coating is as follows: forward average current density > negative current density > GO concentration > negative duty cycle > forward duty cycle; when taking the average coefficient of friction as the evaluation index, the priority order of the influence of each parameter on the coefficient of friction is as follows: forward average current density > negative duty cycle > negative current density > GO concentration > forward duty cycle. By comprehensively analyzing the microhardness and average friction coefficient, the optimal combinations of process parameters were determined as follows: the concentration of GO is 1.0 g/L, the forward current density is 0.50 A/dm^2^, the negative current density is 0.08 A/dm^2^, the forward duty cycle is 65%, and the negative duty cycle is 40%. The Ag-G composite coating prepared under these parameters showed a microhardness of 142.775 HV_0.2_ and an average friction coefficient of 0.506.

On the basis of orthogonal tests, the effects of GO concentration, forward/negative average current density and forward/negative duty cycle on the surface quality, mechanical and tribological properties of the coatings were also systematically investigated by a single-factor experiment. The results demonstrated that process parameters significantly influence the coating quality. A forward average current density of 0.45 A/dm^2^ ensured both a sufficient deposition rate and a dense coating structure. In contrast, a negative average current density of 0.08 A/dm^2^ contributed to grain refinement and facilitated the in situ reduction in GO. A forward duty cycle of 67.5% provided adequate deposition time, while a negative duty cycle of 32.5% improved coating uniformity via a moderate negative dissolution effect. Additionally, a GO concentration of 0.75 g/L yielded an optimal balance between G dispersion and content within the coating.

### 3.3. Coating Properties

#### 3.3.1. Microscopic Morphology of Coatings

**Figure 3:**
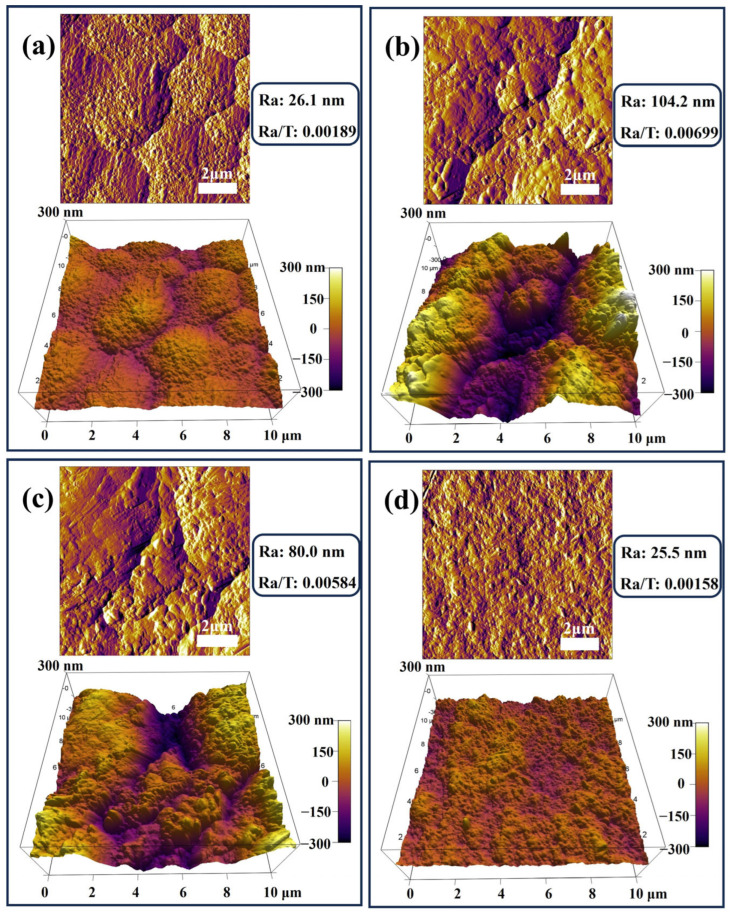
AFM images of four types of coatings: (**a**) #1; (**b**) #2; (**c**) #3; (**d**) #4.

The surface roughness of conductive plating layers is a key factor affecting their performance. Lower roughness enables more uniform current distribution, enhancing conductivity and wear resistance, while also being critical to the performance and reliability of microelectronic devices. The preparation method decisively influences roughness. Compared to direct current plating, pulse plating techniques—such as dual-pulse plating—effectively suppress dendrite formation by precisely controlling nucleation and growth, yielding denser, flatter coatings [40]. The microstructural characterization of the coating in this study was initially performed using AFM for the quantitative analysis of surface morphology and roughness (Ra). Figure 3 shows the surface morphology of four different coatings and their Ra values, and the surface differences and roughness variations in each coating can be clearly observed from the 3D reconstruction of AFM. Coating #1 (Figure 3a) has a flat and dense surface with an Ra value of 26.1 nm, with little surface undulation and small height variation in the region. Coating #2 (Figure 3b) has a rough surface with an Ra value of 104.2 nm, with obvious peaks and valleys structure and uneven surface. This inhomogeneous surface topography originates from the phenomenon of abnormal grain growth or particle agglomeration due to the agglomeration of G during the deposition process, which can lead to the degradation of coating mechanical properties and corrosion resistance. Coating #3 (Figure 3c) has an Ra value of 80.0 nm and a roughness between coatings #1 and #2, with a relatively uniform surface and reduced fluctuations. Coating #4 has the optimum surface flatness with an Ra value of 25.5 nm, slightly better than the coating #1. It has the smallest range of height variation and the fewest surface defects. Additionally, for conductive coatings, surface flatness typically exhibits a positive correlation with performance. A lower ratio of roughness to thickness (Ra/T) indicates a smoother coating surface. As shown in the data, all four coatings exhibit relatively low ratios, with coating #4 displaying the smallest value, indicating its optimal surface flatness.

**Figure 4:**
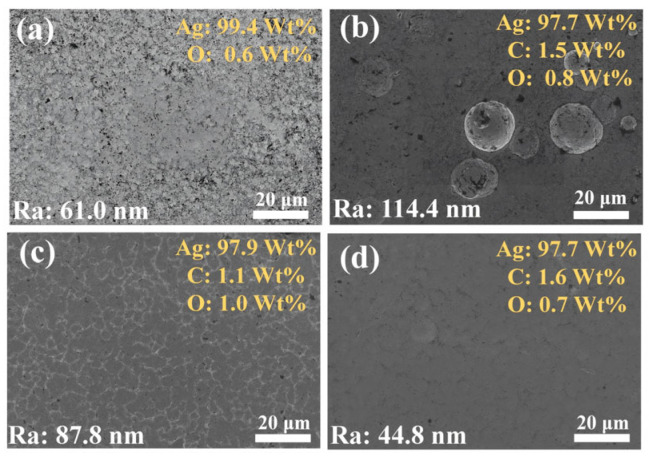
SEM images and EDS analysis of the surface morphology of four coatings: (**a**) #1; (**b**) #2; (**c**) #3; (**d**) #4.

Subsequently, this study used SEM equipped with EDS to characterize the morphology of the coating. As seen from the SEM image (Figure 4), the #1 coating has holes on the surface and is not structurally dense. Large silver particles are distributed on the surface of #2 coating, resulting in a significantly higher roughness than the other coatings, which is due to the abnormal growth of silver particles caused by G agglomeration and uneven distribution. The surface of the #3 coating has no obvious silver particles, but there are still undulations and the surface flatness needs to be improved. In contrast, the #4 coating has a flat, dense surface with minimal roughness, demonstrating the significant effect of the optimized double-pulse electrodeposition process in improving the coating quality. According to the EDS analysis, carbon elements were not identified in the #1 coating, while C elements were present on the surfaces of the #2, #3, and #4 coatings, which proves the successful introduction of G into the coatings. In particular, the O-element content of the #2 coating is close to that of the #3 and #4 coatings, which suggests that the oxygen-containing functional groups of GO were partially removed in the strongly alkaline plating environment and electrodeposition process, and thus reduced to G with fewer defects and introduced into the coating [41,42].

**Figure 5:**
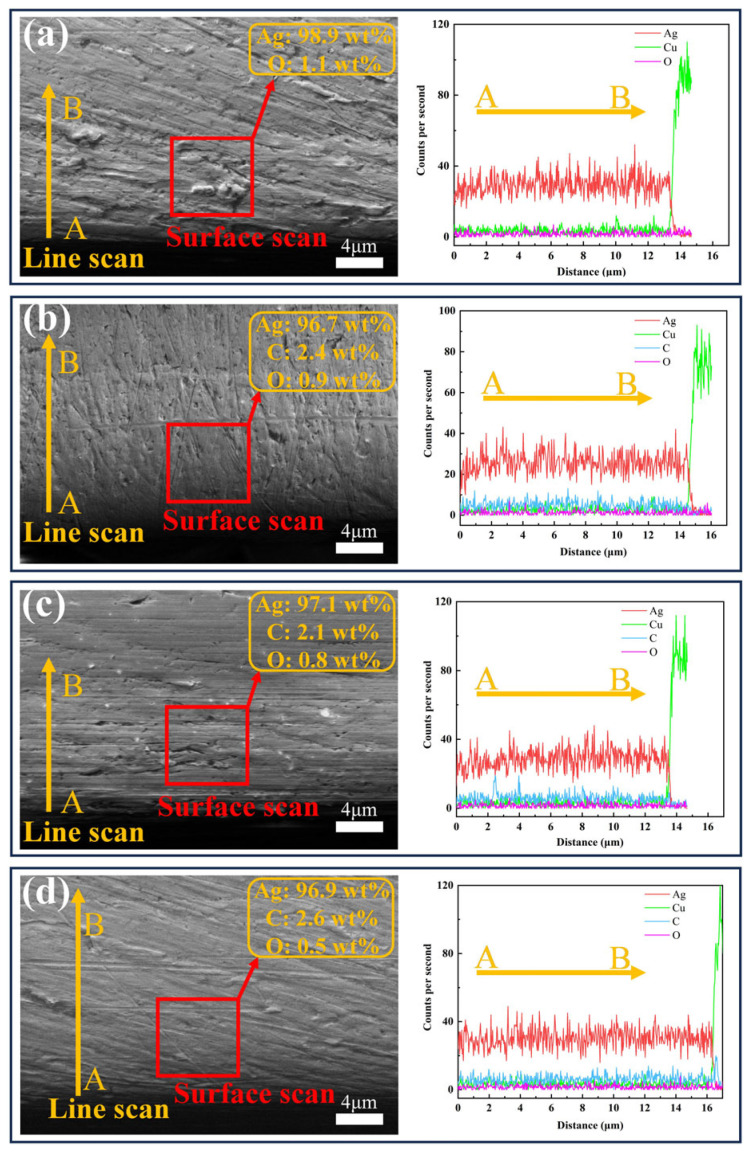
SEM images and EDS analysis of the cross-sections of four coatings: (**a**) #1; (**b**) #2; (**c**) #3; (**d**) #4.

SEM and EDS analysis of the coating cross-sections (Figure 5) revealed strong adhesion between all coatings and the copper substrate, with no interfacial gaps or defects observed. EDS surface and line scanning results demonstrated an absence of carbon in coating #1, while coatings #2–#4 contained detectable carbon. This finding, consistent with surface EDS data, verifies G incorporation in these coatings. The #4 coating has the highest content of C element, indicating that the double-pulse process effectively improves the coating quality. O was detected in all four coatings, with the lowest O in coating #4, indicating that the GO has been reduced to G. EDS line scan results also indicate that the thicknesses of coatings 1–4 are 13.8 μm, 14.9 μm, 13.7 μm, and 16.1 μm, respectively. This confirms that all coating thicknesses meet the experimental requirements.

#### 3.3.2. Characterization of Graphene in Coatings

**Figure 6:**
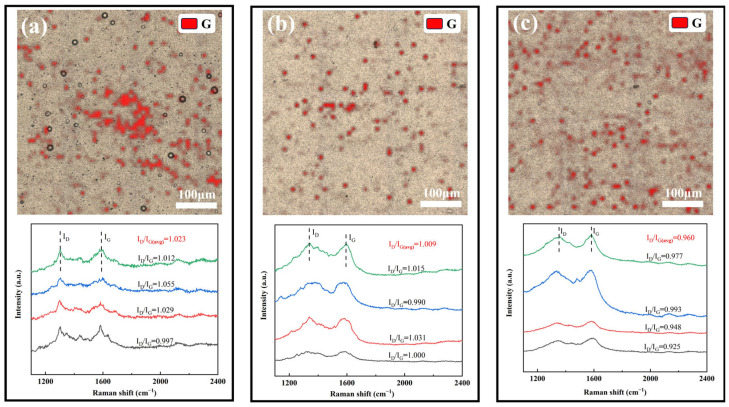
Raman scanning results of three coating surfaces: (**a**) #2; (**b**) #3; (**c**) #4.

In this study, the distribution of G in the composite coatings under three different process conditions, #2, #3 and #4, was systematically characterized by Raman spectroscopic surface scanning. A representative area of 500 μm× 500 μm on the coating surface was randomly selected for the experiment, Surface Raman mapping was performed on a randomly selected 500 μm × 500 μm coating area using a 53 × 53 point scanning array. The G distribution was visualized by tracking the intensity of its characteristic G peak (~1580 cm^−1^), with the resulting mapping data presented in Figure 6 for all three coatings, which show that although the overall G content of the #2 coating prepared by conventional DC electrodeposition is high, its distribution exhibits obvious localized aggregation characteristics, which suggests that there is obvious agglomeration of G in the plating solution under the conditions of the conventional electrodeposition process and non-covalently modified G, resulting in poor dispersion homogeneity of it in the final coating. In contrast, the #3 coating prepared after covalent modification of G exhibited more continuous and uniform G distribution characteristics. On the basis of the #3 coating, the #4 coating prepared by the double-pulse electrodeposition process not only exhibits optimal distribution uniformity, but also a significantly higher G content. This is attributed to two aspects: One is the improvement of the dispersion stability of covalently modified G (GO) in the plating solution, and the other is the more controllable process of GO being partially reduced to G during the double-pulse electrodeposition process.

In order to quantify the structural quality of G, this study characterizes the defect density of G by calculating the I_D_/I_G_ values in the Raman spectra of the four coatings. The results show that the average values (I_D_/I_G (avg)_) of I_D_/I_G_ for coatings #2, #3, and #4 are 1.023, 1.009, and 0.960, respectively, with a gradual decreasing trend. The decreasing of the I_D_/I_G_ values indicates that the defect density of G is gradually reduced, which proves that GO undergoes an effective reduction reaction in the electrodeposition process. The double-pulse electrodeposition process can more thoroughly remove the oxygen-containing functional groups in GO, significantly improve its reduction degree [41], and effectively inhibit the agglomeration and stacking of G.

**Figure 7:**
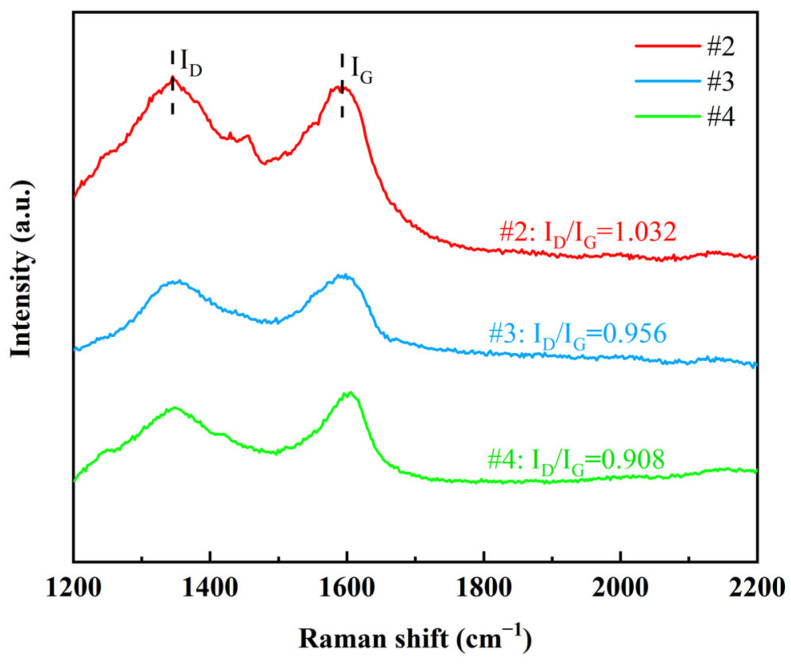
Raman spectra of graphene cross-sections of three coatings.

In order to verify whether G is effectively embedded inside the coatings, Raman spectroscopy was performed in this study on the cross-sections of the three coatings #2, #3, and #4 (Figure 7). The results showed that the D peak (~1350 cm^−1^) and G peak (~1580 cm^−1^) were detected for all three coating cross-sections, indicating that the characteristic structure of G was preserved inside the coating, and the embedding of G in the metal matrix was successfully realized. By quantitatively analyzing the I_D_/I_G_ values, it was found that the #2 coating had the highest I_D_/I_G_ value, indicating that the non-covalently modified G suffered from large structural damage during the embedding process and the presence of agglomeration phenomenon, which led to its high defect density. In contrast, the lower I_D_/I_G_ value for coating #3 indicates that GO undergoes a reduction reaction during the electrodeposition process and is embedded in the coating with a lower defect density. The lowest I_D_/I_G_ value for the #4 coating demonstrates that the double-pulse electrodeposition process significantly improves the degree of reduction and distribution uniformity of GO, resulting in a higher quality of G with fewer defects. In summary, the double-pulse electrodeposition process demonstrated obvious advantages in enhancing the dispersion, reduction degree and structural integrity of G.

#### 3.3.3. Coating Mechanical Properties and Grain Size

**Figure 8:**
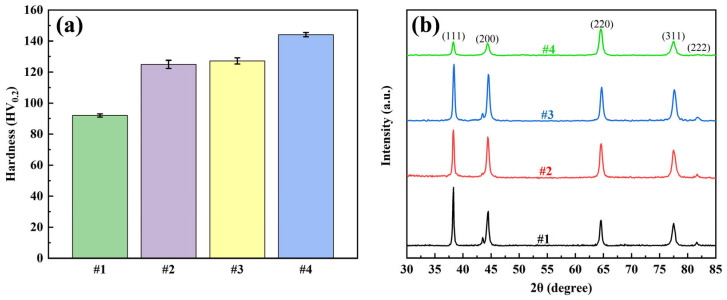
Four coatings: (**a**) microhardness test results and (**b**) XRD test results.

The mechanical properties of the coatings were evaluated by microhardness and XRD tests, and the results are shown in Figure 8. The hardness test showed that the microhardness of the #1 coating was 92.0 HV_0.2_, and the microhardness of the #2 coating was enhanced to 124.9 HV_0.2_, but the agglomeration and inhomogeneous distribution of G limited the performance enhancement. The microhardness of the #3 coating was slightly higher at 127.2 HV_0.2_, while the microhardness of the #4 coating using the double-pulse process was significantly increased to 144.1 HV_0.2_. This enhancement is mainly attributed to the homogeneous dispersion of GO in the plating solution, where the double-pulse electric field promotes the reduction in GO and its uniform distribution into the coating. The introduction of G enhances the strength of the composite coating and promotes grain refinement by hindering silver grain growth and providing more nucleation sites for silver ions.

The XRD results showed that all four coatings showed typical diffraction peaks of silver, which indicated that the addition of G had no significant effect on the basic crystal structure of silver. The grain size was further calculated by the Debye-Scherrer formula, and the results are listed in Table 4. The grain size of coating #1 was the largest at 32.1 nm. The grain size of coating #2 decreased compared to that of #1, but the decrease was not significant, which may be due to the poor dispersion of G in the plating solution, which led to the agglomeration of G and did not sufficiently refine the grains. In contrast, the grain size of coating #3 decreased significantly, indicating that the good dispersion of GO and the reduction reaction helped to refine the grains. The grain size of #4 coating was further reduced to 19.4 nm using double-pulse power supply, which was significantly better than that of #3 coating, this indicates that the negative current of the double pulse current further refines the grain structure.

According to the Hall-Petch relationship, the yield strength or hardness of a material is inversely proportional to the square root of its grain size. The results of this study are highly consistent with this theory: Coating #1, with the largest grain size (32.1 nm), exhibited the lowest hardness (92.0 HV_0.2_); whereas Coating #4, with the smallest grain size (19.4 nm), demonstrated the highest hardness (144.1 HV_0.2_). Grain refinement is primarily attributed to the introduction and uniform distribution of graphene: on one hand, graphene acts as a hetero-nucleation site promoting silver grain nucleation; on the other hand, its physical hindrance effect inhibits grain growth. The dual-pulse process further enhances this refinement effect by optimizing graphene dispersion and reduction degree, resulting in a pronounced Hall-Petch relationship between grain size and hardness. This confirms grain refinement as the key mechanism for coating strengthening.

#### 3.3.4. Tribological Properties of Coatings

**Figure 9:**
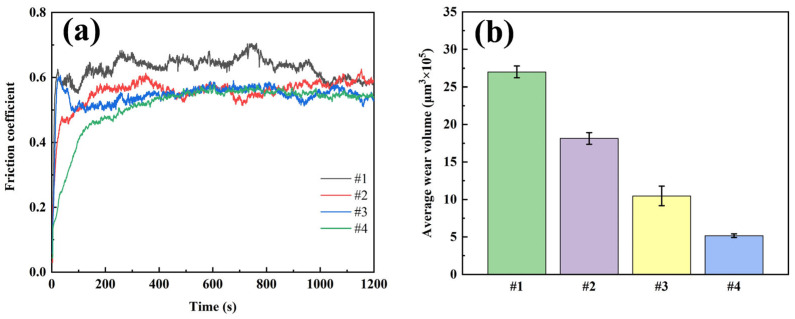
Dry friction test results of four coatings: (**a**) friction curve; (**b**) average wear volume.

Dry friction tests were conducted on four coatings using UMT, and the morphology of the wear points was measured using a white-light interferometric three-dimensional topography scanner. The volume of the wear traces was calculated, and the results are shown in Figure 9. The friction coefficient test shows that the #1 coating has the highest friction coefficient (0.626) and fluctuates a lot, which shows poor friction performance; the friction coefficient of the #2 coating decreases after the addition of G but the friction curve still fluctuates a lot due to the uneven distribution of G; the friction coefficient of coating #3 is similar to that of coating #2, but it is more stable. This is due to improved uniformity of G distribution, but the friction coefficient remains high because the reduction degree of G is still insufficient; Coating #4 has the lowest and most stable coefficient of friction, indicating that the dual-pulse process effectively improves the thinning degree and uniformity of G distribution. Wear volume test results show that coating #4 has the smallest wear volume, 80.9% smaller than coating #1, proving its excellent wear resistance.

These results show that GO was reduced to G under the action of a strongly alkaline plating solution and a bidirectional electric field, and was uniformly distributed in the coating, playing the dual roles of reinforcing agent and lubricant. During friction, exfoliated G particles formed a uniform lubricating film at the contact interface, minimizing direct surface contact. This self-lubricating mechanism led to a substantial decrease in friction coefficient and wear rate, thereby preventing severe wear damage.

**Figure 10:**
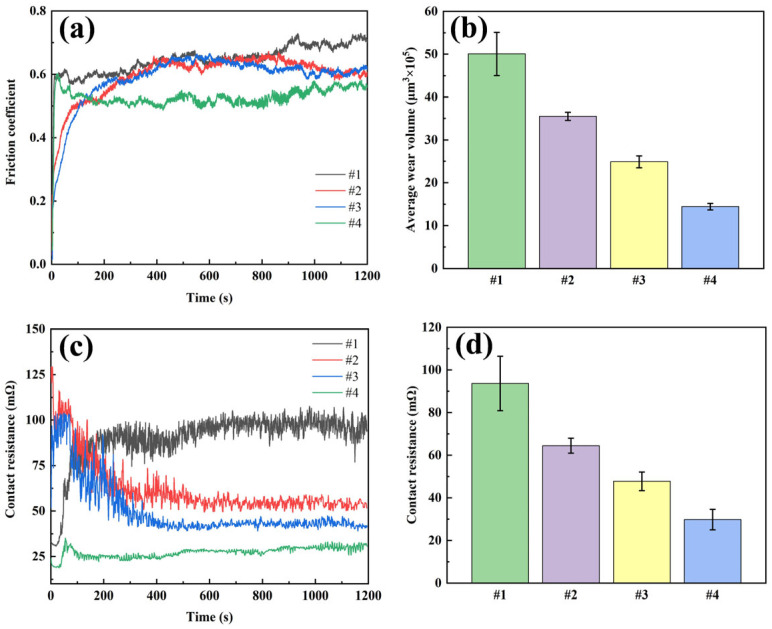
Current-carrying friction test results of four coatings: (**a**) friction curve; (**b**) average wear volume; (**c**) contact resistance curve; (**d**) average contact resistance.

In this study, the friction performance, resistance characteristics and wear of the four coatings were tested by simulating the actual electrical contact conditions through current-carrying friction experiments (Figure 10). The friction coefficients of all coatings increased significantly and fluctuated after the introduction of current. The friction coefficient of coating #1 was the highest (0.650) and showed drastic fluctuations, mainly due to the presence of severe material transfer at the friction interface, which was also confirmed by the SEM images of the wear marks. For the #2 coating with G addition, the friction coefficient decreased to 0.596, but the friction curve still had intermittent spikes due to the agglomeration and uneven distribution of G. Coating #3 using DC deposition in Ag-GO plating solution has a similar friction coefficient as coating #2, but the smoothness of the friction curve is improved, which is attributed to the improved dispersion of G. The #4 coating using the double-pulse power process exhibited optimal performance with the lowest friction coefficient and the smallest curve fluctuation, confirming the role of the double-pulse electric field in promoting the reduction and uniform embedding of GO.

The wear volume showed that the #1 coating had the largest wear volume (50.1 × 10^5^ μm^3^), the #2 and #3 coatings had 35.5 × 10^5^ μm^3^ and 25.0 × 10^5^ μm^3^, respectively, while the #4 coating had the smallest wear volume (14.4 × 10^5^ μm^3^), which was 71.3% lower than coating #1. Among them, the average contact resistance and its fluctuation of the #4 coating were the smallest among the four coatings, which was attributed to the uniformly distributed G in the coating: on the one hand, the G provided lubrication and friction reduction; on the other hand, it suppressed the arc erosion, which significantly improved the electrical contact performance of the coating.

**Figure 11:**
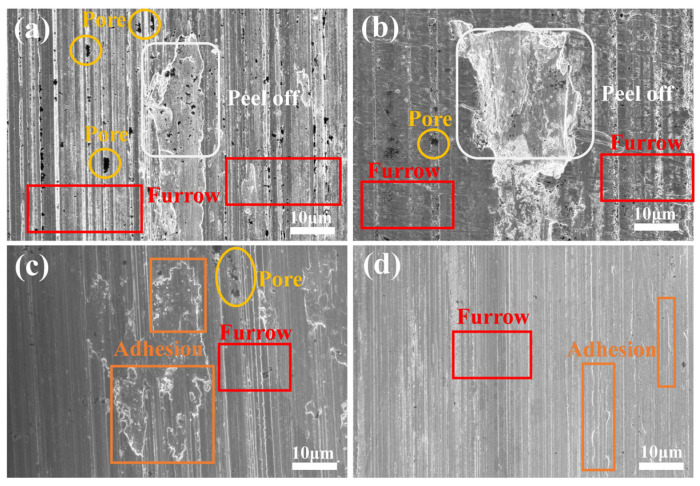
Wear scars of four types of coatings under current-carrying friction experiments: (**a**) #1; (**b**) #2; (**c**) #3; (**d**) #4.

Figure 11 illustrates the SEM images of the abrasion marks of the four coatings after the current-carrying friction experiments. The abrasion marks of the #1 coating show severe furrows and extensive peeling, which indicates that significant abrasive wear has occurred. Due to the softness of the sterling silver material, it is prone to material spalling, and the spalled material acts as abrasive particles in friction, further aggravating the wear. In addition, the abrasion marks are densely distributed with holes, indicating that material loss occurred during friction as a result of arc erosion. Areas of porosity, peeling, and furrowing were present in the wear marks of coating #2, the number of pores was reduced compared to coating #1, but localized peeling was present. The peeling areas were more pronounced and deeper, suggesting that the addition of G improved the material hardness but resulted in deeper wear marks due to the uneven distribution, leading to localized stress concentrations. The wear marks of the #3 coating contain only a small number of holes and shallow furrows, with a significant reduction in the number of pores, but adhesive wear occurs. This suggests that the uniform distribution of G inhibited material loss and interfacial failure, but large patches of adhesive wear still occurred due to the poor reduction in G. The abrasion marks of the #4 coating showed only a small amount of shallow furrowing and adhesion areas with the surface. This indicates that the pulsed process further optimized the reduction and distribution of G, which significantly improved the wear resistance and interfacial stability of the material. The localized adhesion areas may be due to the lubricating film formed by G during the friction process, which resists the erosive effect of the arc and further reduces the coefficient of friction and wear rate.

#### 3.3.5. Corrosion Resistance of Coatings

**Figure 12:**
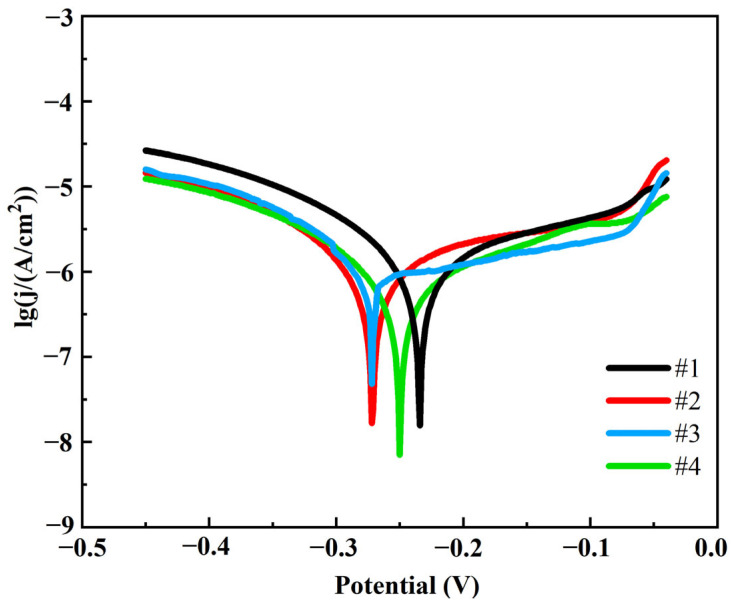
Tafel polarization curves of four coatings.

Figure 12 illustrates the kinetic potential polarization curves of the four coatings in 3.5% NaCl corrosion solution. In order to compare the corrosion resistance of different coatings, the autocorrosion potential (E_corr_), corrosion current density (I_corr_), corrosion rate (CR), anodic slope (b_a_) and cathodic slope (b_c_) were calculated by Tafel fitting, and the specific results are shown in Table 5.

As can be seen in Table 5, coating #4 has the lowest corrosion current density and slowest corrosion rate, which is 45.5% lower than coating #1. In contrast, coating #1 had the highest corrosion current density and corrosion rate, indicating the worst corrosion resistance. The #2 and #3 coatings performed between the #1 and #4 coatings, with the #3 coating having a significantly lower corrosion current density and corrosion rate than the #2 coating due to the uniform distribution of G. This indicates that the double-pulse power process significantly improves the corrosion resistance of the coatings. This is mainly attributed to the following two reasons: firstly, the pulsed current promotes the uniform distribution and reduction in G, which enhances the densification of the coating and reduces the penetration path of the corrosive medium; secondly, the G is able to inhibit the corrosive reaction effectively by virtue of its high chemical stability and barrier properties to the corrosive medium. At the same time, the flatness and denseness of the coating surface structure reduces the anodic dissolution area and blocks the penetration of corrosive media. The coatings prepared by the double-pulse electrodeposition process show more excellent corrosion resistance due to the higher G content and better surface quality.

#### 3.3.6. Electrical Contact Life of Coatings

An electrical life test was conducted on an electrical contact material tester to evaluate the enhanced performance of the four coatings under actual working conditions. The contact resistance was monitored in real time during the experiments, and the change curve of contact resistance with the number of on–offs was plotted. The failure criterion of the coating is whether there is a sudden and large surge in contact resistance.

**Figure 13:**
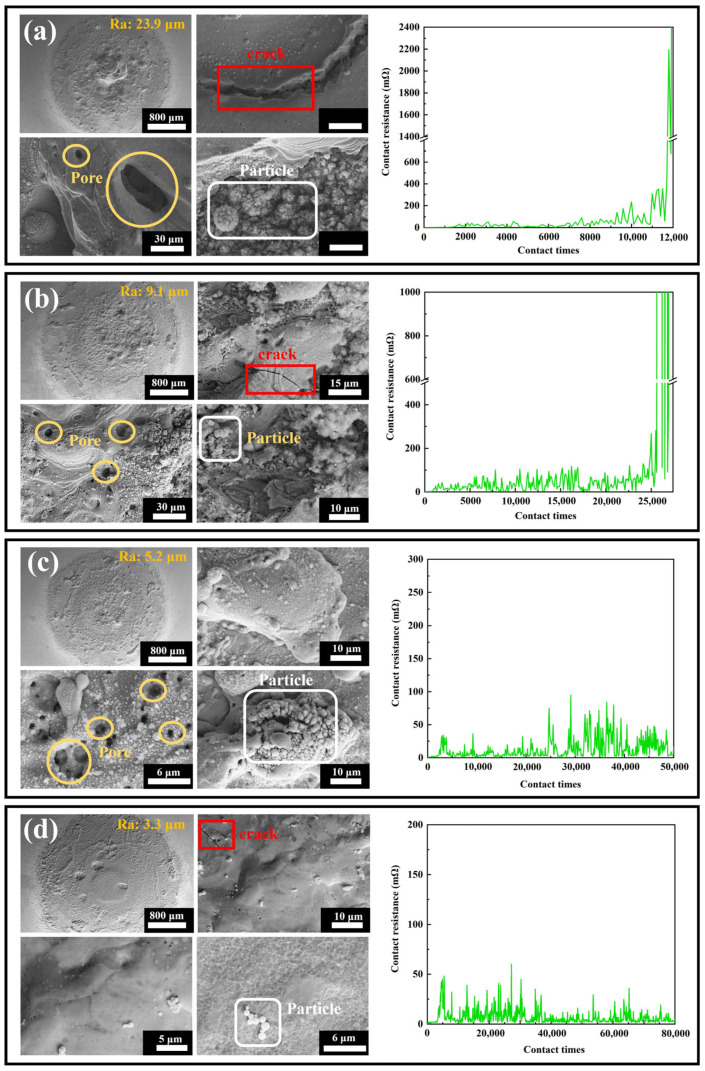
Overall and local scanning electron microscope images of the contact surfaces after electrical life testing, along with real-time resistance curves during electrical life testing, for the four coatings: (**a**) #1; (**b**) #2; (**c**) #3; (**d**) #4.

Figure 13 illustrates the variation in contact resistance during the electrical contact life test for the four coatings. The contact resistance of coating #1 showed several significant peaks during the test, especially near 2000, 4000 and 10,000 passes. The large fluctuations in the resistance of this coating indicate that it is less stable during the electrical contact life test and may suffer from poor localized contact or non-uniform material properties. Eventually, at about 11,500 passes, the contact resistance spiked to 2000 mΩ, indicating that the Ag coating on the contact surface had lost its protective effect and the material had failed. Coating #2 behaved similarly to coating #1, with significant peaks in contact resistance near 5000, 10,000, and 15,000 passes, but the number and magnitude of the peaks were relatively small. The contact resistance of coating #3 remained stable within 100 mΩ after 50,000 passes, and the fluctuation of resistance was small throughout the test, with only slight fluctuations during a few passes, showing good stability. After 80,000 passes, the contact resistance of coating #4 was consistently stabilized within 50 mΩ, and there was almost no significant peak in the resistance value during the test, with only minor fluctuations in a few passes, showing excellent stability and durability.

The thermal stability of electrical contact materials is a critical factor determining their reliability, fault tolerance, and service life under high currents, arc impacts, and repetitive operations. Materials with insufficient thermal stability are prone to melting, evaporation, oxidation, or structural deformation due to high arc temperatures, leading to increased contact resistance, welding, or mechanical failure. This study systematically evaluated the thermal stability of Ag-G (silver–graphene) composite coatings through electrical contact life testing combined with SEM surface morphology analysis. Figure 13 displays the overall and local microstructures of the four coatings after testing, with the following results: Coating #1 (base silver layer) exhibited a surface roughness as high as 23.9 μm, featuring a coarse structure with prominent cracks and uneven pores. Local images reveal long, deep cracks originating from the silver layer’s low hardness, which is prone to fatigue failure under repeated electrical contact and mechanical stress. Voids primarily formed from localized material melting or evaporation due to high arc temperatures. Numerous spherical particles were also visible, formed by surface tension after material melting. This coating exhibited poor thermal stability with weak resistance to arc erosion and thermal fatigue. #2 coating (graphene-dispersed but unevenly distributed) showed overall roughness reduced to 9.1 μm, with a smoother yet still coarse structure. Local morphology reveals reduced crack quantity, length, and depth, with more uniform pore distribution and decreased spherical particle count and size. Graphene introduction enhances coating hardness and thermal conductivity, improving resistance to arc erosion and heat transfer. However, its uneven distribution still causes localized stress and heat concentration, inducing minor cracks, pores, and spherical particles. This indicates improved thermal stability, though still constrained by dispersion uniformity. #3 Coating (uniform graphene distribution but insufficient reduction) exhibits significantly reduced surface roughness to 5.2 μm with a smoother structure. Cracks are nearly eliminated, and spherical particle count and size further decrease, though pore count slightly increases-likely due to incomplete graphene reduction. Uniformly distributed graphene effectively suppresses crack propagation and localized melting, markedly improving thermal stability, though structural defects persist from insufficient reduction. #4 Coating (dual-pulse optimized process, uniformly distributed graphene with high reduction) exhibited the lowest roughness (3.3 μm) and the smoothest, densest structure. Local images revealed virtually no pores, only minimal microcracks and spherical particles, with extremely few surface defects. The optimized process significantly improved graphene distribution uniformity and reduced agglomeration, greatly enhancing coating density and interfacial stability. This coating exhibits outstanding thermal stability, demonstrating the strongest resistance to arc erosion, melting, and evaporation. In summary, the thermal stability of Ag-G coatings strongly depends on the uniformity of graphene distribution and reduction degree. Optimizing the double-pulse electrodeposition process effectively improves graphene dispersion and reduction, thereby significantly enhancing the coating’s overall performance and service life.

## 4. Conclusions

To overcome the performance limitations of composite electrical contact materials caused by the uneven dispersion of the reinforcing phase, this study proposes a dual optimization strategy that simultaneously optimizes the silver plating solution system and the electroplating process. This strategy successfully achieves uniform distribution of G in the silver matrix. The synergistic optimization results in the preparation of high-performance coatings, and their effectiveness is verified at both the microscopic and macroscopic levels. The main conclusions are as follows:

(1) In the study of regulating the ζ value in silver plating solutions, the introduction of oxygen-containing functional groups onto the surface of covalently modified graphene (GO) significantly increased the |ζ| value of the silver plating solution, thereby significantly improving the dispersion stability of G.
(2) The dispersion of G in the composite coating was further regulated by optimizing the electrodeposition process parameters. The optimal process parameters were determined through a comprehensive analysis of the orthogonal and single-factor experiment: the concentration of GO is 0.75 g/L, the average forward current density is 0.45 A/dm^2^, the average negative current density is 0.08 A/dm^2^, the forward duty cycle is 67.5%, and the negative duty cycle is 32.5%.
(3) In the performance study of Ag-G composite coatings, covalently modified graphene (GO) was combined with a double-pulse process to reduce GO to G under the action of a strongly alkaline plating solution and a bi-directional electric field, which was uniformly distributed in the coating. Microstructural analysis showed that the coating surface was dense and smooth with a grain size of 19.4 nm, and the G was uniformly distributed with low defect density. Mechanical property tests showed that the microhardness of the coating was 144.1 HV0.2, which was 56.6% higher than that of the pure Ag coating. Tribological performance studies showed that the coating exhibited the lowest coefficient of friction and wear under both dry and current-carrying friction conditions. Corrosion resistance tests showed that the corrosion current density and corrosion rate of the coating were reduced by 45.5% compared to the pure Ag coating, respectively. In the electrical contact life test, the contact resistance of the coating was stabilized within 50 mΩ after 80,000 cycles, showing excellent stability and durability.

## Figures and Tables

**Table 1:** Plating solution composition and plating parameters for four coatings.

Composition and Conditions	#1	#2	#3	#4
Common Components and Parameters	AgNO_3_: 45 g/L, C_6_H_5_NO_2_: 100 g/L, CH_3_COONH_4_: 76 g/L, K_2_CO_3_: 72 g/L, KOH: 45 g/L, NH_3_·H_2_O: 32 mL/L; Temperature: 30 ± 1 °C, pH: 9.5–10.5, Electrolyte agitation: 260 rpm, Deposition thickness: 15 ± 2 μm.			
C_12_H_25_NaO_4_S (g/L)	/	0.030	/	/
C_18_H_29_NaO_3_S (g/L)	/	0.038	/	/
(C_6_H_9_NO)_n_ (g/L)	/	0.023	/	/
Graphene (g/L)	/	0.75	/	/
Graphene oxide (g/L)	/	/	0.75	0.75
Forward average current density (A/dm^2^)	0.26	0.26	0.26	0.45
Negative average current density (A/dm^2^)	/	/	/	0.08
Forward duty cycle (%)	/	/	/	67.5
Negative duty cycle (%)	/	/	/	32.5

**Table 2:** Results and range analysis of the orthogonal test for three additives.

Group	SDS (wt%)	SDBS (wt%)	PVP (wt%)	|ζ| (mV)
1	3	3	3	12.38
2	3	4	4	8.52
3	3	5	5	3.72
4	4	3	4	12.52
5	4	4	5	6.83
6	4	5	3	17.29
7	5	3	5	7.78
8	5	4	3	11.27
9	5	5	4	6.32
Range analysis				
n_1_	8.21	10.89	13.65	/
n_1_	12.21	8.87	9.12	/
n_1_	8.46	9.11	6.11	/
R	4.00	2.02	7.50	/
Priority	PVP > SDS > SDBS			
Optimal level	4	3	3	10.61

**Table 3:** Orthogonal test results and range analysis.

Group	C_GO_ (g/L)	J_f_ (A/dm^2^)	J_r_ (A/dm^2^)	D_f_ (%)	D_r_ (%)	HV (HV_0.2_)	COF
1	0.5	0.20	0.04	65.0	30.0	122.963	0.557
2	0.5	0.30	0.06	70.0	35.0	126.275	0.532
3	0.5	0.40	0.08	75.0	40.0	131.275	0.520
4	0.5	0.50	0.10	80.0	45.0	128.175	0.584
5	1.0	0.20	0.06	75.0	45.0	127.100	0.573
6	1.0	0.30	0.04	80.0	40.0	134.438	0.532
7	1.0	0.40	0.10	65.0	35.0	127.650	0.567
8	1.0	0.50	0.08	70.0	30.0	141.813	0.510
9	1.5	0.20	0.08	80.0	35.0	122.250	0.572
10	1.5	0.30	0.10	75.0	30.0	124.500	0.565
11	1.5	0.40	0.04	70.0	45.0	130.213	0.577
12	1.5	0.50	0.06	65.0	40.0	139.113	0.532
13	2.0	0.20	0.10	70.0	40.0	116.500	0.600
14	2.0	0.30	0.08	65.0	45.0	128.025	0.580
15	2.0	0.40	0.06	80.0	30.0	129.688	0.534
16	2.0	0.50	0.04	75.0	35.0	134.338	0.527
HV range analysis							
n_1_	127.172	122.203	130.488	129.438	129.741	/	/
n_2_	132.750	128.310	130.544	128.700	127.628	/	/
n_3_	129.019	129.707	130.841	129.303	130.332	/	/
n_4_	127.138	135.860	124.206	128.638	128.378	/	/
R	5.612	13.657	6.635	0.800	2.704	/	/
Priority	J_f_ > J_r_ > C_GO_ > D_r_ > D_f_						
Optimal level	1.0	0.50	0.08	65.0	40.0	142.775	0.506
COF range analysis							
n_1_	0.548	0.576	0.548	0.559	0.542	/	/
n_2_	0.546	0.552	0.543	0.555	0.550	/	/
n_3_	0.562	0.550	0.546	0.546	0.546	/	/
n_4_	0.560	0.538	0.579	0.556	0.579	/	/
R	0.016	0.038	0.036	0.013	0.037	/	/
Priority	J_f_ > D_r_ > J_r_ > C_GO_ > D_f_						
Optimal level	1.0	0.50	0.06	75.0	30.0	136.288	0.541

**Table 4:** Average grain size of four coatings.

Coating	2θ (°)	β (°)	D (nm)
1	38.276	0.259	32.1
2	38.289	0.332	25.1
3	38.383	0.358	23.3
4	38.300	0.429	19.4

**Table 5:** Data corresponding to the Tafel polarization curves of four coatings.

Coating	Ecorr (V)	Icorr (μA∙cm^−2^)	ba (V∙dec^−1^)	bc (V∙dec^−1^)	CR (mm∙year^−1^)
1	−0.2281	1.4250	3.7780	−7.1965	0.04795
2	−0.2674	1.3351	2.8988	−6.7222	0.04492
3	−0.2322	0.9521	3.1855	−6.5103	0.03203
4	−0.2359	0.7771	5.0600	−6.7887	0.02614

## Data Availability

The data presented in this study are available upon reasonable request from the corresponding author.
